# Neuroinflammation-informed neuroimaging-transcriptomic signatures explaining acupuncture’s therapeutic effects in chronic insomnia

**DOI:** 10.1186/s13020-025-01236-5

**Published:** 2025-11-28

**Authors:** Wenting Lin, Liyong Yu, Hao Xu, Xiangwen Xiao, Zihao Xia, Zeyang Dou, Daijie Hu, Yuqi He, Lili Yang, Jie Yang, Tianmin Zhu, Fang Zeng, Siyi Yu

**Affiliations:** 1 School of Rehabilitation and Health Preservation, Chengdu University of Traditional Chinese Medicine, Chengdu, China; 2 Chengdu Pidu District Hospital of Traditional Chinese Medicine/The Third Affiliated Hospital of Chengdu University of Traditional Chinese Medicine (West District), Chengdu, China; 3 School of Acupuncture and Tuina, Chengdu University of Traditional Chinese Medicine, No.37 Shierqiao Road, Chengdu, 610075 Sichuan China; 4 School of Medical Imaging, North Sichuan Medical College, Nanchong, China; 5 Key Laboratory of Acupuncture for Senile Disease (Chengdu University of TCM), Ministry of Education, Chengdu, China

**Keywords:** Chronic insomnia disorder, Acupuncture, Neuroinflammation, Global brain connectivity, Imaging transcriptomics

## Abstract

**Background:**

Chronic insomnia disorder (CID) is characterized by dysregulation in brain function and is closely associated with neuroinflammation. Although acupuncture has been shown to improve insomnia symptoms, its underlying mechanisms, particularly at both the macro brain connectivity and corresponding molecular levels, remain unclear

**Methods:**

Forty-eight CID patients were randomly assigned to either an acupuncture group or a waitlist group. Clinical data and resting-state functional magnetic resonance imaging (rs-fMRI) data were collected before and after the intervention. Changes in brain connectivity were analyzed using fMRI to assess global brain connectivity (GBC) in each group. Gene expression data from the Allen Human Brain Atlas were utilized to identify important genes contributing to these acupuncture-induced GBC changes. Gene set enrichment analysis was performed to annotate the molecular biological processes involved.

**Results:**

In the acupuncture group, fMRI analysis revealed decreased regional GBC in key regions, such as the pallidum and prefrontal cortex, correlating with symptom relief. In contrast, the waitlist group showed increased regional GBC without symptom relief. Gene set enrichment analysis revealed that specific genes associated with astrocytes and neuroinflammation-related biological processes were linked to the acupuncture-induced changes in GBC. The neuroinflammation-informed GBC-transcriptomic signatures induced by acupuncture were further validated by their significant correlation with reductions in IL-6 levels as insomnia symptoms improved.

**Conclusion:**

Acupuncture may remodel brain functional connectivity by regulating neuroinflammation-related pathways, thereby improving insomnia symptoms.

**Graphical abstract:**

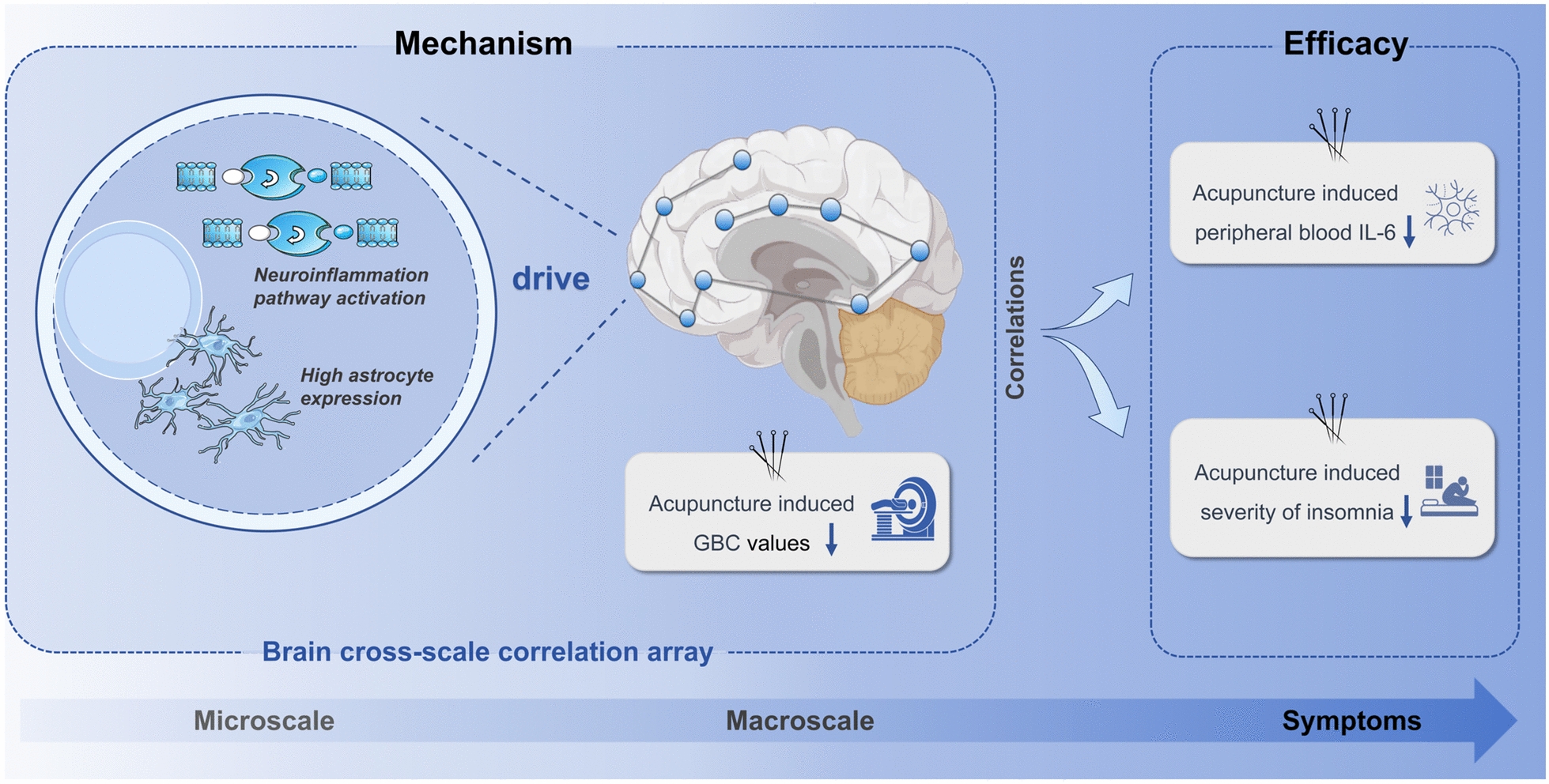

**Supplementary Information:**

The online version contains supplementary material available at 10.1186/s13020-025-01236-5.

## Introduction

Chronic insomnia disorder (CID) is a prevalent sleep disorder characterized by persistent difficulty in initiating or maintaining sleep, leading to impaired daytime functioning and reduced quality of life [1–3]. Acupuncture, a traditional Chinese medicine therapy, has demonstrated clinical effectiveness in alleviating insomnia symptoms [4–6]. Previous studies suggest that acupuncture may exert therapeutic effects through modulation of inflammatory processes and restoration of aberrant brain connectivity [7, 8]. For instance, at the molecular level, acupuncture has been shown to significantly reduce peripheral inflammatory cytokines, such as interleukin-6 (IL-6) and tumor necrosis factor-alpha (TNF-α), thereby mitigating inflammatory responses [9–11]. At the macroscopic level, acupuncture treatments have been associated with enhanced functional connectivity between brain regions involved in emotion regulation and sleep–wake control, such as the prefrontal cortex, amygdala, and hypothalamus [12–14]. However, despite these findings, the field still lacks an integrated understanding of the cross-scale mechanisms—that is, how acupuncture-induced macro-level changes in brain connectivity are linked to micro-level molecular and cellular processes. Addressing this gap is essential for elucidating the biological pathways through which acupuncture exerts its therapeutic effects. Recent advances in neuroimaging–transcriptomic analysis provide unique opportunities to bridge this knowledge gap and explore these multi-level interactions [15].

Resting state functional magnetic resonance imaging (re-fMRI) is a valuable tool for investigating brain connectivity patterns associated with various treatments, including acupuncture [16, 17]. Among fMRI-based analyses, global brain connectivity (GBC) is particularly effective for capturing the broad-scale alterations in neural communication across the brain [18, 19]. Our previous study, including one using EEG-derived connectivity, showed that patients with CID exhibit abnormal GBC in key regions such as the medial prefrontal cortex (mPFC), and that these alterations can serve as predictive biomarkers [20]. Although EEG and rs-fMRI differ in temporal resolution, GBC in both modalities quantifies correlated activity patterns between brain regions to index large-scale network organization, with rs-fMRI providing higher spatial resolution for precise anatomical localization. These advantages make rs-fMRI-derived GBC a valuable and spatially detailed indicator for evaluating treatment efficacy and for providing potential mechanistic insights into how acupuncture may modulate brain function to alleviate insomnia symptoms.

The development of neuroimaging-transcriptomic analysis and the availability of the Allen Human Brain Atlas (AHBA) have significantly advanced our capability to dissect brain function at a molecular level [21–23]. Utilizing AHBA gene expression data enables researchers to associate regional changes in brain activity with specific gene patterns [22]. Our previous study using neuroimaging-transcriptomic analysis bridged the gap between cortical structural alterations and underlying molecular mechanisms in CID patients, highlighting that molecular pathways such as chronic neuroinflammation may contribute to the observed abnormalities in structural connectivity [24]. Additionally, this integrated analysis has been successfully applied to investigate the mechanisms of other treatments, such as electroconvulsive therapy for depression, where changes in brain structure were linked to alterations in gene expression related to neuroplasticity [25], further demonstrating its potential to elucidate the treatment mechanisms for various neurological disorders [19]. However, it remains unclear whether neuroinflammation-related abnormalities evolve with clinical improvement in CID. This integration of transcriptomic insights with neuroimaging data offers a promising avenue for identifying molecular pathways underlying the macro-scale changes induced by acupuncture.

**Fig. 1:**
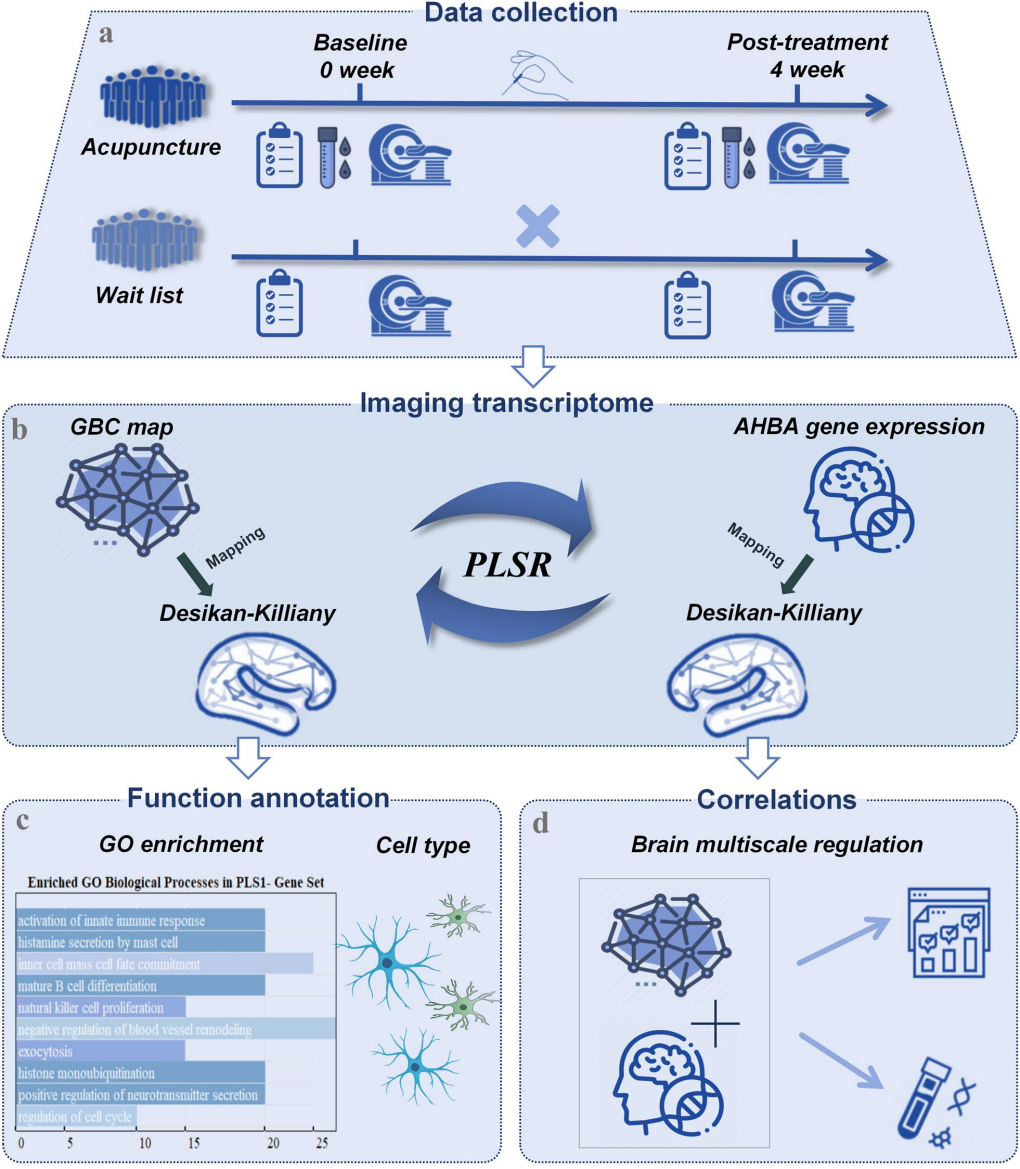
Guidelines for the study. **a** Summary of data collection in each group, detailing the types of data (fMRI, clinical scales, peripheral inflammation markers) collected at each visit. **b** Imaging transcriptomics analysis, illustrating the association between acupuncture-induced changes in macroscopic global brain connectivity and microscopic gene expression patterns. **c** Decoding brain imaging changes from a molecular perspective, including gene ontology enrichment and cell-type enrichment analysis. **d** Association of acupuncture-induced brain cross-scale modulation with the reduction of peripheral pro-inflammatory factors and improvement of insomnia symptoms

In this study, we hypothesize that acupuncture-induced improvements in insomnia symptoms result from modulation of neuroinflammation-related pathways, reflected by changes in GBC. To test this hypothesis, we collected clinical and resting-state fMRI data before and after acupuncture treatment for CID, and we included a waitlist group as a control. We then analyzed changes in GBC and employed partial least squares regression (PLS-R) analysis to explore corresponding gene expression patterns from the AHBA. Moreover, gene set enrichment analysis was performed to determine whether the inflammation biological processes were involved. Additionally, peripheral blood inflammatory markers were collected before and after acupuncture treatment to validate the role of acupuncture in modulating inflammation-related pathways. The overview of the study is illustrated in Fig. 1. By combining fMRI-derived connectivity measures with transcriptomic data from AHBA, we aim to elucidate the cross-scale biological mechanisms by which acupuncture exerts therapeutic effects for CID.

## Methods

### Study design

This was a randomized, neuroimaging-based, controlled trial conducted in accordance with neuroimaging guidelines [26] and the Standards for Reporting Interventions in Clinical Trials of Acupuncture (STRICTA) guidelines [27]. Building on previous studies utilizing acupuncture treatment for insomnia disorder [28, 29], our aim was to validate the hypothesis that acupuncture can improve clinical symptoms of CID by modulating neuroinflammation-related brain functional changes. We chose to include a waitlist group instead of a sham acupuncture group, as many acupuncture clinical trials have reported placebo effects in the sham acupuncture group, but not evident in the waitlist group [30, 31]. We speculate that both the sham acupuncture group and the true acupuncture group may have therapeutic effects on insomnia patients. Due to the need for a more reliable comparison between the acupuncture intervention and the absence of treatment, we opted for an acupuncture group and a waitlist group rather than including a sham acupuncture group. The acupuncture group received a standardized acupuncture regimen, while the waitlist group did not receive treatment until after the study period. Clinical evaluations and resting-state fMRI (rs-fMRI) were performed on all participants at the beginning and end of the study. Additionally, the acupuncture group also provided peripheral blood samples to measure inflammatory markers (Fig. 1). The study received ethical approval (approval number: 2021KL-125) from the Ethics Committee of the Affiliated Hospital of Chengdu University of Traditional Chinese Medicine (CDUTCM) and was registered with the Chinese Clinical Trial Registry (ChiCTR, https://www.chictr.org.cn) under the registration number ChiCTR2200058878.

### Participants

Patients with CID were enrolled in the outpatient clinics of the Affiliated Hospital of CDUTCM and Chengdu Second People’s Hospital from April 2022 to November 2022. The diagnosis of CID was conducted through structured interviews by three neurologists with 8–15 years of psychiatric experience, based on the International Classification of Sleep Disorders, Third Edition (ICSD-3) [32]. All participants signed an informed consent form before participating in the study. Participants were eligible for inclusion if they were right-handed individuals of any gender, aged between 18 and 60 years; scored > 7 on the Pittsburgh Sleep Quality Index (PSQI); experienced insomnia symptoms (including difficulty falling asleep, maintaining sleep, or early awakening) at least three times per week for more than three months; and had not taken any hypnotic drugs or other psychiatric medications within two weeks prior to enrollment. Exclusion criteria included a history of consciousness disorders; other sleep disorders such as sleep apnea syndrome, sleep-related movement disorder, central disorders of hypersomnolence, and circadian rhythm sleep–wake disorders; severe cardiac, hepatic, renal, cerebral, or hematopoietic system diseases; a history of mental disorders including depression, bipolar disorder, anxiety, schizophrenia, or personality changes; pregnancy or lactation or those intending to become pregnant within the next three months; a history of alcohol, drug, or caffeine dependence; and contraindications for magnetic resonance imaging, such as the implantation of electronic devices or metallic objects.

### Intervention

**Fig. 2:**
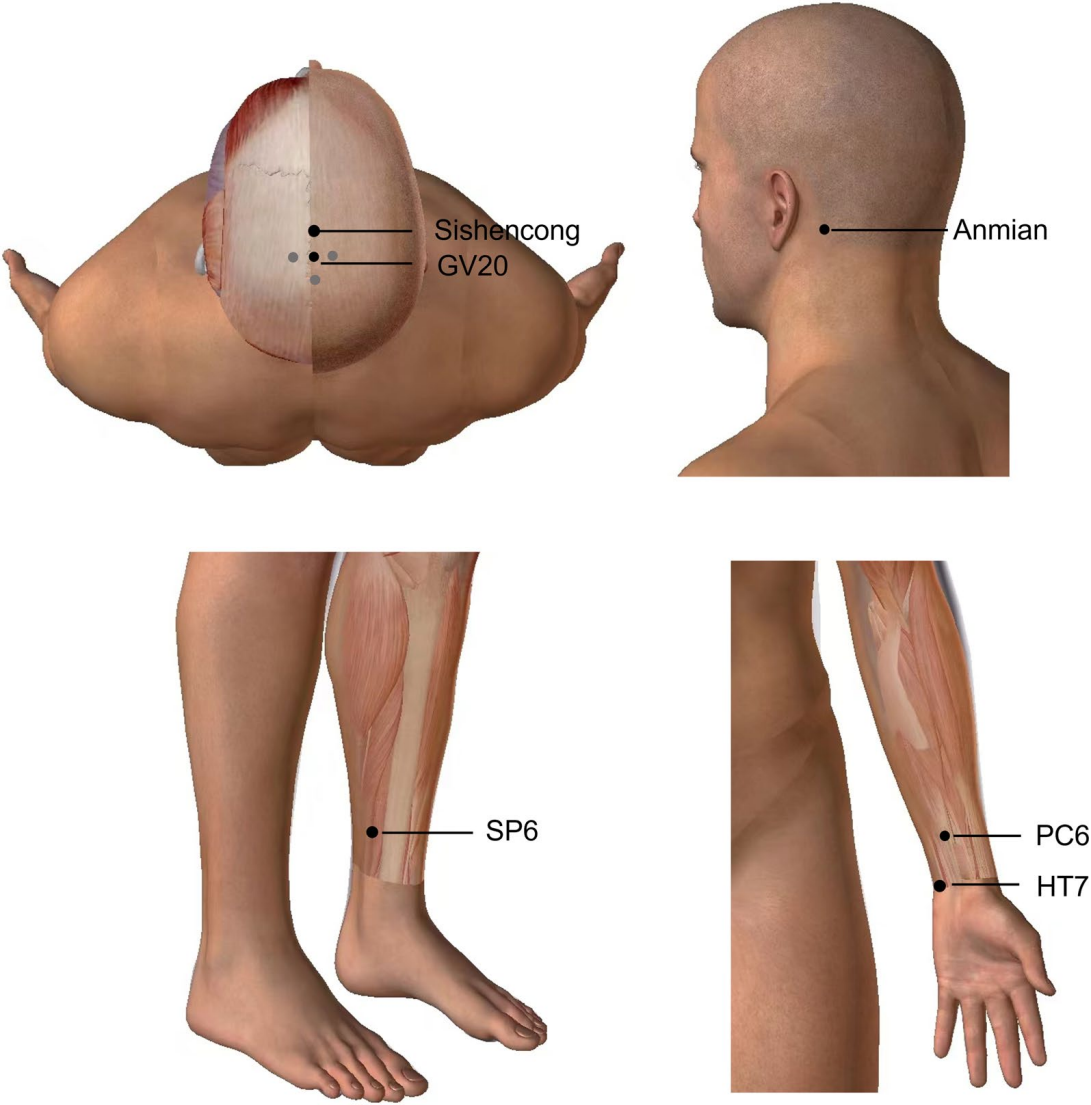
Location of acupoints for acupuncture treatment. Acupoints where stimulation was provided via needles were marked as black points

Acupuncture was administered by two trained, licensed acupuncturists with over three years of clinical experience. The selected acupoints included Baihui (GV20), Sishencong, Anmian, Shenmen (HT7), Neiguan (PC6), and Sanyinjiao (SP6), with bilateral stimulation for all except GV20 and Sishencong (Fig. 2). Disposable stainless-steel needles (0.30 mm in diameter, 25 mm and 40 mm in length) were utilized. The 'Deqi' sensation, a clinical marker of effective acupuncture characterized by sensations of pain, heaviness, numbness, swelling, or radiation, was induced through needle manipulation techniques such as lifting, thrusting, twirling, and rotating. Each session lasted approximately 30 min, with treatments occurring once daily for a total of 20 sessions over four weeks. Participants in the waitlist group did not receive acupuncture treatment during the study period but were assured of receiving 20 compensatory acupuncture sessions after the study’s completion.

### Clinical assessment

At the baseline and end of the 4-week intervention, all patients were evaluated using the PSQI and the Insomnia Severity Index (ISI) to measure sleep quality and the severity of insomnia, respectively. Furthermore, emotional states were assessed using the Self-rating Anxiety Scale (SAS) and the Self-rating Depression Scale (SDS).

### Sample size

The sample size calculation was based on post-treatment PSQI scores and their standard deviations from a prior study [28]. In that study, the acupuncture group had a post-treatment PSQI score of 8.62 ± 2.93, while the control group had a score of 14.76 ± 3.35. To estimate the required sample size, we used the following formula:$N={[\frac{({z}_{\alpha }+{Z}_{\beta })\sigma }{\delta -\Delta }]}^{2}$. where Z_α_ = 1.96 (corresponding to α = 0.05 for a two-tailed test), Z_β_ = 1.28 (for a desired power of 90%), and σ represents the pooled standard deviation calculated from the two groups. The difference in PSQI scores between the acupuncture and control groups was δ = 6.14, and Δ = 3.8 represents the threshold for a clinically meaningful difference [33]. Based on these parameters, the calculation suggested 19 participants per group. After accounting for a 20% dropout rate, the required number of participants per group was adjusted to 24. Therefore, with a 1:1 allocation ratio, a total of 48 participants were included in the study.

### Peripheral blood collection

Patients in the acupuncture group provided blood samples before and after the 4-week treatment period. Fasting venous blood samples were obtained between 8:00 and 9:00 AM on the day of the MRI scan. Whole blood was collected in tubes containing ethylenediaminetetraacetic acid (EDTA) anticoagulant, centrifuged at 3000 rpm for 10 min, and serum samples were stored at -80 °C. Serum levels of IL-6, TNF-α, interleukin-1 beta (IL-1β), and C-reactive protein (CRP) were determined using the Rayto RT-6100 diagnostic instrument and enzyme-linked immunosorbent assay kits.

### Image data acquisition

MRI scans were conducted on a 3.0-Tesla MR system (Discovery MR750w, General Electric, Milwaukee, WI, USA) equipped with a 32-channel head coil. Tight but comfortable foam padding was used to minimize head motion, and earplugs were provided to reduce scanner noise. Sagittal 3D T1-weighted (T1W) images were acquired using a brain volume sequence with the following parameters: repetition time (TR) = 7.06 ms; echo time (TE) = 3.04 ms; flip angle (FA) = 12°; field of view (FOV) = 256 mm × 256 mm; matrix = 256 × 256; slice thickness = 1 mm, no gap; and 188 sagittal slices. The rs-fMRI datasets were obtained in 8 min with a gradient-recalled echo-planar imaging pulse sequence. The rs-fMRI imaging parameters were TR = 2000 ms, TE = 30 ms, FA = 90°, acquisition matrix = 64 × 64, FOV = 100 × 100 mm, thickness = 3.5 mm, voxel size = 3.5 × 3.5 × 4.02 mm^3^, gap = 0.5 mm, and number of slices = 33. A total of 240 volumes were acquired. During the scans, participants were instructed to relax and keep their eyes closed, and all patients reported that they did not fall asleep during the scanning.

### Image data preprocessing

T1w and rs-fMRI data processing were performed on fMRIPrep 22.1.1 pipelines [34]. For the T1w image, a sequence of procedures was conducted, which encompassed intensity non-uniformity correction, skull-stripping, tissue segmentation, and spatial normalization to a standard space through nonlinear registration. The fMRI preprocessing procedures encompassed reference volume generation, head-motion parameter estimation prior to spatiotemporal filtering, slice-time correction, co-registration with the T1w reference, extraction of confounding time series, and resampling into standard MNI152NLin2009cAsym space. The fMRI preprocessing procedures encompassed reference volume generation, head-motion parameter estimation prior to spatiotemporal filtering, slice-time correction, co-registration with the T1w reference, extraction of confounding time series, and resampling into standard space. Additionally, the CONN toolbox [35] (version 22a) based on SPM 12 [36] was employed for various operations, including smoothing with a 6 mm Full-Width at Half-Maximum (FWHM) and regression of nuisance covariates (six motion parameters obtained from the realignment).

### Global brain connectivity analysis

GBC was employed as a macroscopic neuroimaging outcome for each group, given its sensitivity to the effects of interventions in longitudinal studies [18, 19]. GBC was estimated using the CONN toolbox and custom-written code [20]. Initially, for each scan, we calculated the average time series for all voxels within each of the 83 regions of interest (ROIs) defined by the Desikan-Killiany (DK) atlas [37], which encompasses 68 cortical and 15 subcortical areas. These subcortical regions include the bilateral thalamus, caudate, putamen, pallidum, hippocampus, amygdala, accumbens, and brainstem. Subsequently, we determined the pairwise Pearson’s correlation coefficients for the mean time series across all region pairs. Finally, we converted these correlation coefficients into Fisher’s Z values and calculated the mean of all positive values, maintaining all positive correlations without using any threshold. Those processes were used to calculate GBC maps. Consistent with previous recommendations [38–40], negative correlations were excluded from GBC calculations due to the limited biological interpretation of such correlations. To assess treatment effects, we applied paired t-tests to quantify baseline-to-post changes in GBC for each brain region within each group, providing a macro-level measure for subsequent correlation with gene expression profiles. This approach is stable and interpretable [19, 23], as the sign and magnitude of the t-value directly reflect the direction and size of GBC change. To account for multiple comparisons, a false discovery rate (FDR) adjustment was applied to the p-values, considering the total number of regions (n = 83) examined.

### Decoding GBC changes from a molecular perspective

This study initially delineated normative gene expression patterns from the AHBA and mapped these profiles onto the Desikan-Killiany 83 atlas [37]. Due to the availability of right hemisphere data for only two donors (Table S1), the transcriptomic-imaging association analysis was limited to the left hemisphere, encompassing 34 cortical and 7 subcortical regions, and was represented by a 41-region × 12,506 gene matrix. Subsequently, PLS-R [20] was employed to discern the covariance between gene expression patterns and acupuncture-induced GBC changes. PLS-R is particularly suited to imaging–transcriptomics, as it directly extracts gene expression patterns most strongly associated with the observed neuroimaging phenotype, in contrast to unsupervised methods such as principal component analysis [41]. The first component in PLS-R (PLS1) was found to account for the most variance in acupuncture-induced GBC changes. Bootstrapping was used to estimate the Z-score for each gene as its contribution to PLS1 [41]. Furthermore, to interpret PLS-R results, we used gene-category enrichment analysis [42] and cell-type enrichment analysis [43] to uncover which molecular pathways were enriched among the most highly associated genes. Spatial permutation test and FDR correction were used to reduce false positives during enrichment analysis. Details of those analyses can be found in our Supplementary materials.

### Statistical analysis

All analyses were conducted in R version 4.1.0. Clinical outcome indicators (PSQI, ISI, SAS, and SDS) were analyzed using linear mixed-effects models (LMMs) to account for the repeated-measures structure and potential missing data, implemented via the *lme4* package in R. Each model included group (acupuncture, waitlist), time (baseline, post-treatment), and their interaction (group × time) as fixed effects, with a random intercept for each participant to model within-subject correlations. Age and sex were included as covariates in all models to control for potential confounding effects. The primary hypothesis was evaluated through the significance of the group × time interaction, testing whether the magnitude of change from baseline differed between groups. Model assumptions were assessed by visual inspection of residual plots and by testing normality (Shapiro–Wilk), with rank-based LMMs applied as sensitivity analysis if assumptions were violated. For peripheral inflammatory markers (IL-6, IL-1β, TNF-α, CRP), which were collected only in the acupuncture group, within-group changes over time were analyzed using LMMs with time as a fixed effect and a random intercept for each participant. Statistical significance for fixed effects was obtained using Satterthwaite’s approximation with the *lmerTest* package. All p-values were two-tailed, and multiple comparisons were corrected using the Benjamini–Hochberg FDR method where applicable.

To assess the significance of associations across brain regions, we applied spatial autocorrelation-preserving permutation tests (commonly referred to as ‘spin’ tests) [44, 45], with a significance threshold of p_spin_ < 0.05. This method ensures the preservation of the inherent correlational structure of the cortical surface data, offering stringent control against false positives in contrast to conventional random permutation tests [44]. For the statistical significance of the gene-neuroimaging correlations revealed by the PLS-R models, we compared the real-data-derived PLS1 against null spatial distributions. Additionally, for the correlation analysis between two cortical maps, we also conducted spatial-null models to mitigate spatial autocorrelations. Detailed information is available in the Supplementary materials.

To validate our hypothesis that neuroinflammation-informed GBC-transcriptomics is associated with symptom relief in CID patients, we examined the relationship between acupuncture-induced GBC-transcriptomics and changes in peripheral inflammatory markers and clinical symptoms. We first generated acupuncture-induced GBC alteration maps for each patient by calculating the difference between post-treatment and baseline maps. These maps were then correlated with regional PLS1 score maps using Spearman correlation, providing a value that reflects how each participant’s GBC-transcriptomics pattern manifested. This correlation was further analyzed to assess its association with changes in clinical scores (PSQI, ISI, SAS) and peripheral inflammatory markers (from serum samples collected with fMRI data). Partial Spearman correlations were used for this analysis, controlling for sex and age, with statistical significance set at p < 0.05.

## Results

One patient from the acupuncture group and three patients from the waitlist group were excluded due to scheduling conflicts that caused them to withdraw from the study. No participant was excluded due to head movements during the fMRI scan. The final sample comprised 23 patients in the acupuncture group and 21 patients in the waitlist group. Further details on the demographic data are provided in Table 1.

**Table 1:** Demographic and clinical information of each group

Variable	Study group, mean (SD)		Mean difference (95%CI)	p value		
	Acupuncture (n = 23)	Waitlist (n = 21)		Group effect	Time effect	Interaction effect
Female (%)	19.0 (82.6)	13.0 (61.9)		0.18*		
Age (years)	36.8 (9.9)	33.3 (9.9)		0.15^▲^		
PSQI						
Baseline	13.3 (2.5)	12.5 (2.6)	0.9 (− 0.6 to 2.5)			
4 wk	7.3 (2.5)	11.8 (2.1)	− 4.3 (− 5.9 to − 2.8)	0.013	< 0.001	< 0.001
ISI						
Baseline	17.3 (3.9)	15.6 (3.4)	2.2 (− 0.2 to 4.6)			
4 wk	9.2 (4.4)	15.4 (4.0)	− 5.7 (− 8.1 to − 3.3)	0.094	< 0.001	< 0.001
SAS						
Baseline	50.1 (9.6)	45.4 (9.6)	5.8 (− 0.3 to 11.9)			
4 wk	42.5 (7.0)	46.2 (12.6)	− 2.6 (− 8.7 to 3.5)	0.573	0.009	0.001
SDS						
Baseline	51.7 (8.7)	48.0 (10.2)	4.9 (− 0.8 to 10.6)			
4 wk	49.0 (7.4)	48.6 (10.9)	1.6 (− 4.2 to 7.3)	0.223	0.377	0.171
TNF-α						
Baseline	11.0 (1.8)	NA				
4 wk	7.4 (2.5)	NA	− 1.3 (− 2.5 to − 0.2)^a^		0.026^a^	
IL-6						
Baseline	20.1 (1.6)	NA				
4 wk	8.4 (0.9)	NA	− 1.0 (− 1.6 to − 0.4)^a^		0.001^a^	
IL-1β						
Baseline	20.1 (1.6)	NA				
4 wk	15.5 (1.3)	NA	− 4.6 (− 5.4 to − 3.8)^a^		< 0.001^a^	
CRP						
Baseline	2.0 (0.3)	NA				
4 wk	1.90 (0.33)	NA	− 0.1 (− 0.3 to 0.0)^a^		0.128^a^	

^*^Fisher's Exact Test, ^▲^Mann–Whitney U test, ^**a**^Within-group changes over time were analyzed using linear mixed-effects models with time as a fixed effect. *PSQI* Pittsburgh Sleep Quality Index, *ISI* Insomnia Severity Index, *SAS* Self-rating Anxiety Scale, *SDS* Self-Rating Depression Scale, *TNF-α* Tumor Necrosis Factor-alpha, *IL-6* Interleukin-6, *IL-1β* Interleukin-1 beta, *CRP* C-reactive protein, *NA* not applicable

### Effects of acupuncture treatment on clinical symptoms and peripheral cytokines

LMM analysis revealed significant group × time interactions for PSQI, ISI, and SAS scores (all p < 0.001), indicating greater pre-to-post improvement in the acupuncture group compared with the waitlist group (Table 1). Specifically, post hoc contrasts showed that the acupuncture group exhibited substantial reductions in PSQI (estimated mean change [95% CI] = − 4.3 [− 5.9, − 2.8], p < 0.001) and ISI (− 5.7 [− 8.1, − 3.3], p < 0.001), whereas changes in the waitlist group were non-significant (p > 0.05). SAS scores decreased significantly in the acupuncture group (− 2.6 [− 8.7, 3.5], p = 0.001) but not in the waitlist group (p > 0.05), with a significant between-group difference in change. No significant group × time interaction was found for SDS scores (p = 0.171). Since cytokine levels were collected only in the acupuncture group, within-group LMM analysis with time as the fixed effect showed significant post-treatment decreases in IL-6 (− 1.0 pg/mL [− 1.6, − 0.4], p = 0.001), TNF-α (− 1.3 pg/mL [− 2.5, − 0.2], p = 0.026), and IL-1β (− 4.6 pg/mL [− 5.4, − 3.8], p < 0.001), while changes in CRP did not reach statistical significance (p > 0.05, Table 1).

### Macroscopic global brain connectivity at baseline and after acupuncture treatment

**Fig. 3:**
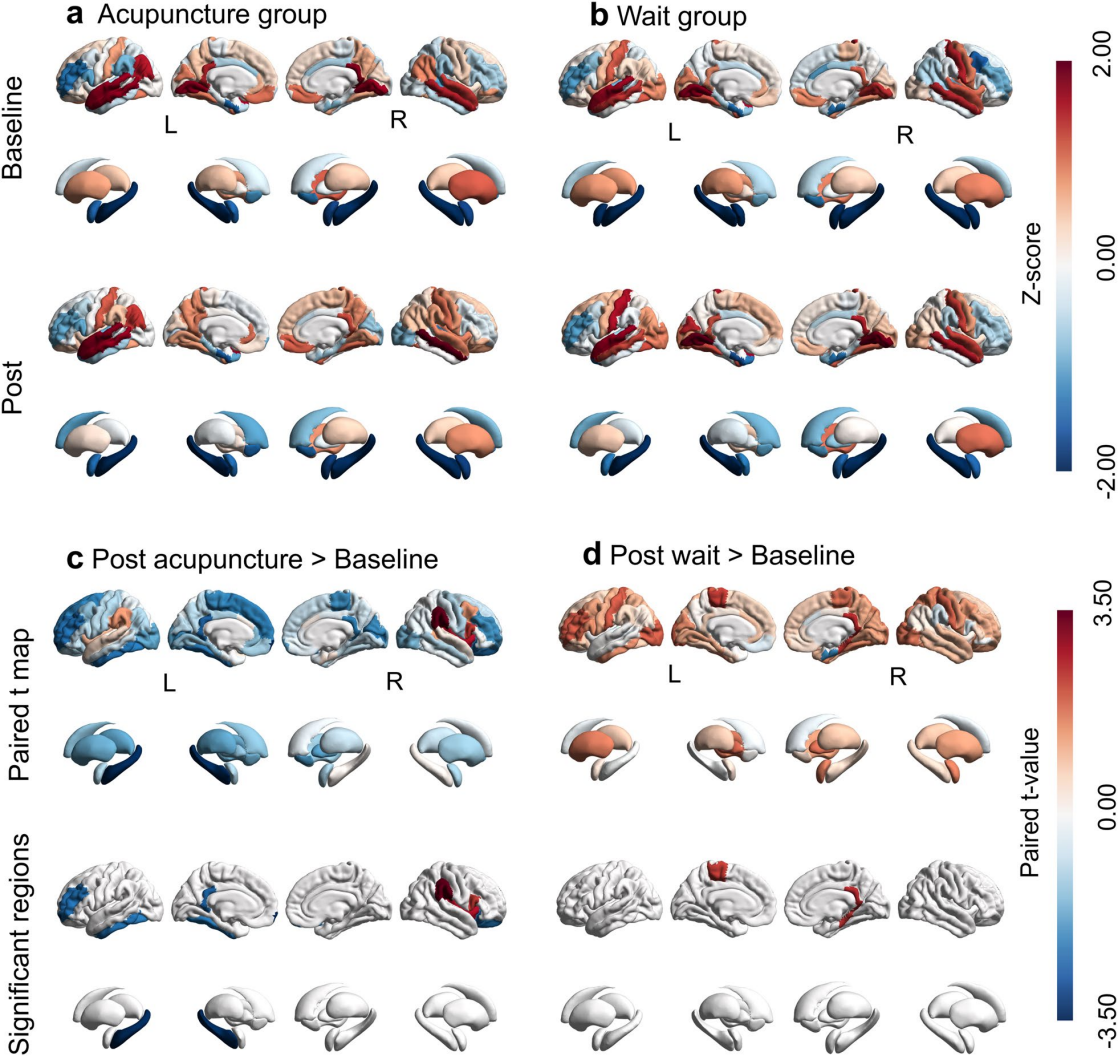
Macroscopic global brain connectivity at baseline and after acupuncture and waitlist treatments. Brain maps depict the regional distribution of changes in GBC in cortical and subcortical regions of the DK atlas. **a** Baseline and post-treatment unthresholded values for the acupuncture group. **b** Baseline and post-treatment unthresholded values for the waitlist group. **c** Within-group changes in GBC for the acupuncture group. **d** Within-group changes in GBC for the waitlist group. For (**c**) and (**d**), the upper row of each panel shows unthresholded paired t-values, and the lower row shows all significant regions at p < 0.05, uncorrected. Colors depict z-scores, where positive (red) and negative (blue) z-scores indicate GBC values above and below the mean GBC, respectively. *DK* Desikan-Killiany, *GBC* global brain connectivity, *L* left, *R* right

**Fig. 4:**
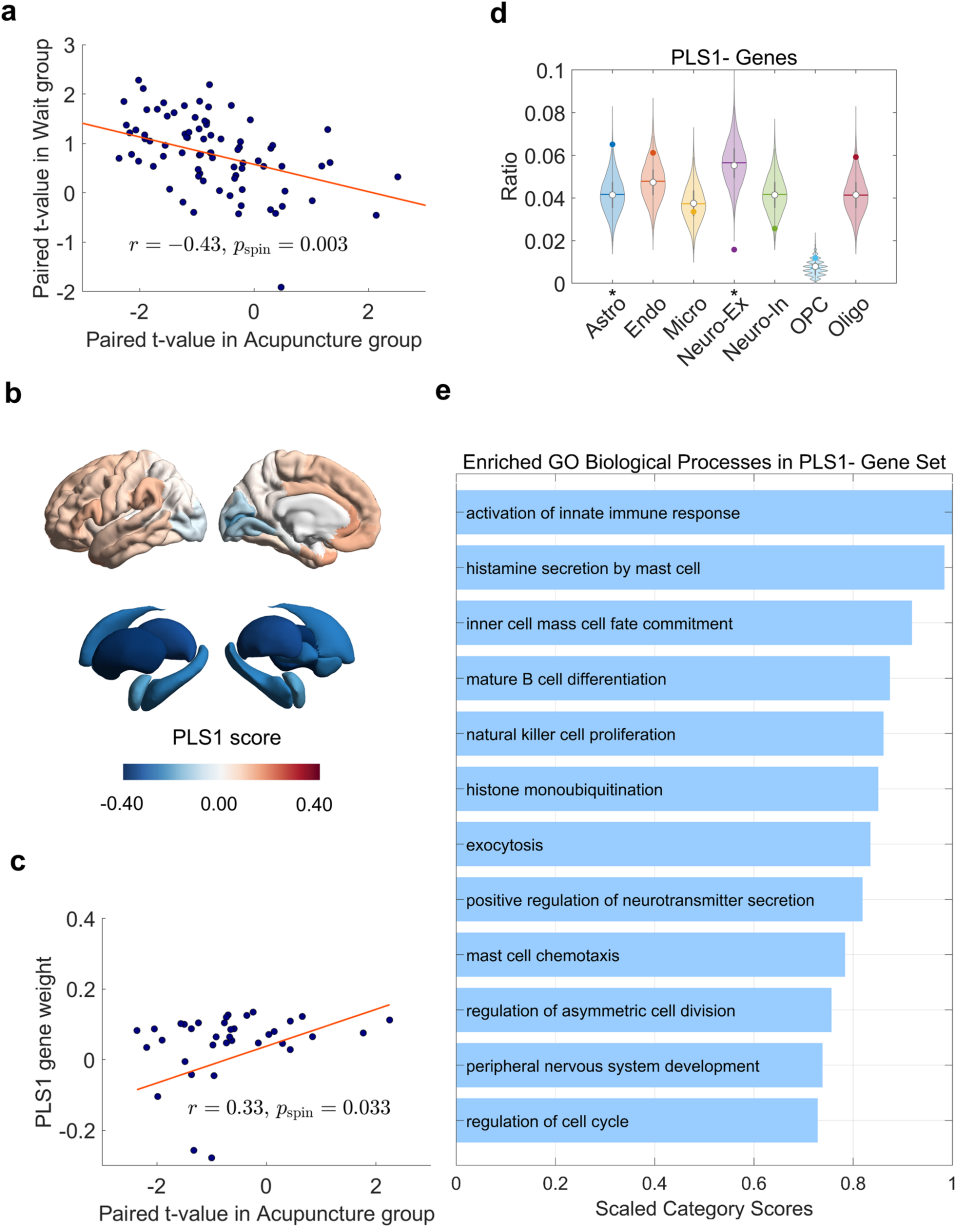
Microscopic transcriptomic vulnerability to acupuncture-induced changes in macroscopic connectivity and the underlying biological and cellular pathways. **a** Spatial patterns of changes in GBC induced by acupuncture and waitlist negatively correlate with each other. **b** Brain maps depict the regional distribution of PLS1 weights across cortical and subcortical regions for acupuncture. **c** The scatter plot shows a positive correlation between the regional distribution of PLS1 weights and the T-statistics quantifying acupuncture treatment-induced changes in GBC. **d** Brain cell-type enrichment. **e** Enrichment for GO – biological pathways terms. The T-statistics of treatment-induced changes in GBC reflect an average over the two brain hemispheres. We only interpreted enrichment for negatively weighted genes. *p_FDR_ < 0.05. *Astro* astrocyte, *DK* Desikan-Killiany, *Endo* endothelial, *GBC* global brain connectivity, *GO* gene ontology, *L*, Left, *Micro* microglia, *Neuro-ex* excitatory neurons, *Neuro-in* inhibitory neurons, *OPC* oligodendrocyte precursor, *Oligo* oligodendrocyte, *PLS* Partial Least Square, *R* right

To facilitate the presentation of GBC values across brain regions before and after treatment in both groups, we performed a Z-transformation of the GBC values for each brain region. The acupuncture group predominantly exhibited decreases in GBC patterns, while the waitlist group dominantly induced increases in GBC (Fig. 3a; Fig. 3b). Specifically, after four weeks of acupuncture treatment, significant decreases in regional GBC were observed in several areas, including the left cingulate isthmus, left frontal pole, left pallidum, and orbitofrontal cortex (Fig. 3c; p < 0.05, uncorrected; Table S2). Conversely, the waitlist group showed significant increases in regional GBC in areas such as the right cingulate isthmus, right parahippocampal gyrus, and left paracentral cortex after the same duration (Fig. 3d; p < 0.05, uncorrected; Table S2). As expected, the spatial patterns of GBC changes induced by acupuncture were negatively correlated with those of the waitlist group (r = − 0.43, p_spin_ = 0.0034, Fig. 4a), supporting the notion that acupuncture may initiate a unique mechanism for modulating GBC patterns compared to the waitlist.

### Microscopic gene expression patterns associated with acupuncture-induced changes in global brain connectivity

For the acupuncture group, PLS1 explained the highest proportion of GBC changes (12.5%) (Fig. 4b) and was significantly positively correlated with regional changes in GBC after acupuncture treatment (r = 0.33; p_spin_ = 0.033) (Fig. 4c). Given that acupuncture-induced changes in GBC were predominantly decreases, the positive correlations between regional PLS1 weights and GBC changes indicate that genes with negative weights are highly expressed in regions where GBC decreased the most and have low expression in regions with small or negligible increases in GBC. Conversely, genes with positive weights are highly expressed in regions where GBC had small or negligible increases and have low expression in regions with the most significant decreases in GBC. Therefore, the potential microbiological factors for macroscopic GBC value changes are likely to be associated with the negatively weighted genes (PLS1- gene sets), which may more accurately represent the underlying biological mechanisms responsible for the acupuncture-induced reduction in GBC.

### Gene enrichment analyses unveil biological and cellular pathways underlying acupuncture-induced macroscopic connectivity alterations

TNAK, a representative gene among the negatively weighted genes (Z = − 3.75, ranking 12,478/12,506), encodes a protein that interacts with tumor necrosis factor receptor-associated factors (TRAFs), thereby influencing multiple immune and inflammation-related signaling pathways [46]. For the acupuncture PLS1 component, we observed significant enrichment of genes with negative weights highly expressed in astrocytes (p_FDR_ < 0.05, Fig. 4d and Table S3). Additionally, we identified significant enrichment for several GO biological pathway terms broadly related to neuroinflammation (Fig. 4e and Table S4), such as “Activation of Innate Immune Response,” “Histamine Secretion by Mast Cell,” and “Mature B Cell Differentiation.”

### Acupuncture-induced GBC-transcriptomics correlates with improvement in peripheral inflammatory responses and insomnia symptoms

The acupuncture-induced GBC-transcriptomics patterns were positively correlated with clinical improvement, specifically (i) a decrease in peripheral IL-6 levels after four weeks of acupuncture treatment and (ii) a reduction in ISI scores after four weeks of treatment (both p < 0.05, Table 2). This finding supports the relationship between acupuncture-induced GBC-transcriptomics changes and peripheral inflammation levels when insomnia symptoms are alleviated.

**Table 2:** Spatial alignment of macroscopic connectivity changes with transcriptome susceptibility patterns correlates with changes in peripheral inflammation markers and clinical symptoms in the acupuncture group

	variable	Rho	*p*-value
Clinical symptoms	∆PSQI	0.067	0.772
	∆ISI	0.477	0.029
	∆SAS	− 0.345	0.126
Peripheral inflammation markers	∆TNF-α	− 0.196	0.394
	∆IL-6	0.479	0.028
	∆IL-1β	− 0.116	0.617
	∆CRP	− 0.150	0.516

Summary of correlations between the extent to which each patient manifests the spatial pattern of the first component of our partial least squares regression model (PLS1) in their pattern of global brain connectivity changes induced by acupuncture and changes in clinical symptoms and peripheral inflammatory markers. *PSQI* Pittsburgh Sleep Quality Index, *ISI* Insomnia Severity Index, *SAS* Self-rating Anxiety Scale, *TNF-α* Tumor Necrosis Factor-alpha, *IL-6* Interleukin-6, *IL-1β* Interleukin-1 beta, *CRP* C-reactive protein

## Discussion

Our findings revealed three key insights into the cross-scale mechanisms underlying acupuncture treatment for CID. First, acupuncture significantly alleviated clinical symptoms in patients with CID, reduced peripheral blood levels of the inflammatory marker IL-6, and decreased GBC in several regions, including the pallidum and prefrontal cortex. Second, the acupuncture-induced reductions in GBC spatially corresponded to brain areas with high expression of genes enriched in astrocytes and biological processes related to neuroinflammation. Third, these neuroinflammation-informed GBC-transcriptomic signatures were further validated by their significant correlation with reductions in IL-6 levels as insomnia symptoms improved. Together, these findings provide cross-scale evidence linking acupuncture's anti-inflammatory effects to functional brain changes, offering a potential mechanistic foundation for its therapeutic role in chronic insomnia.

### Changes in clinical symptoms and peripheral pro-inflammatory factors after acupuncture treatment

Acupuncture, as a millennia-old Chinese therapy, has demonstrated clear therapeutic effects on sleep disorders [47]. It has shown efficacy in improving sleep quality and alleviating anxiety symptoms associated with insufficient sleep [48–50]. Our current findings are consistent with previous studies [48–50], validating acupuncture’s beneficial effects on sleep regulation. In the acupuncture group, PSQI, ISI, and SAS scores significantly decreased (p < 0.05), while no significant changes were observed in the waitlist control group. These clinical improvements highlight acupuncture’s effectiveness in alleviating insomnia symptoms and improving emotional well-being in CID patients. Such results further strengthen the clinical evidence supporting acupuncture as a reliable, non-pharmacological treatment option for chronic insomnia disorder.

Additionally, our study found that acupuncture treatment significantly reduced peripheral blood inflammatory markers, specifically IL-6 and IL-1β (p < 0.05). This finding aligns with previous research demonstrating acupuncture’s capacity to modulate inflammation and thereby exert therapeutic effects [9–11]. For example, acupuncture has been reported to alleviate inflammation by activating the cholinergic anti-inflammatory pathway [51], which suppresses pro-inflammatory cytokine release via vagus nerve stimulation [52]. Furthermore, studies indicate acupuncture reduces neuroinflammation by modulating toll-like receptor (TLR) signaling pathways, thereby inhibiting downstream inflammatory cascades involving NF-κB activation and subsequent cytokine production [53]. Both preclinical and clinical investigations have consistently observed elevated peripheral blood levels of IL-6, IL-1β, and TNF-α in insomnia patients [10, 54]. Supporting these findings, acupuncture interventions have effectively normalized inflammatory markers; for instance, clinical studies have shown significant reductions in peripheral IL-6 and TNF-α levels following acupuncture treatments [9]. Consistent with this evidence, our findings further confirm acupuncture’s dual beneficial impact—relieving clinical symptoms and reducing systemic inflammation—in CID patients.

### Changes in GBC after acupuncture treatment

We found that after 4 weeks of acupuncture treatment, the GBC value decreased significantly in certain brain regions, including the basal ganglia (specifically the pallidum) and parts of the prefrontal lobe (e.g., the frontal pole and orbitofrontal lobe), which are particularly susceptible to neuroinflammation [55]. This supports the hypothesis that acupuncture may exert its therapeutic effects on CID by modulating brain regions linked to neuroinflammation. The pallidum, as a component of the basal ganglia, is implicated in several neurotransmitter release and regulation processes [56]. When inflammation occurs, the pallidum may increase the demand for dopamine, thus affecting neurotransmitter homeostasis [57]. Previous studies [58] have confirmed changes in the volume of the pallidum following electroconvulsive therapy in depressed patients, suggesting that this volume change may be related to inflammatory responses. The prefrontal lobe occupies a central position in the brain; studies have shown that neuroinflammation may directly affect the function and structure of these prefrontal regions [59, 60]. For example, inflammation can disrupt functional connectivity between the lateral prefrontal lobes and other regions [59], and morphological changes in the orbital frontal lobe of fibromyalgia patients have been associated with neuroinflammation [60]. However, despite finding within-group differences in brain connectivity changes between pre- and post-acupuncture treatment that may be related to neuroinflammation, we have not yet provided direct molecular-level evidence.

By contrast, after 4 weeks, the waitlist group and the acupuncture group showed different patterns of change in GBC before and after the trial. Specifically, the waitlist group showed elevated GBC values in a few brain regions, such as the isthmus cingulate cortex, parahippocampal gyrus, and paracentral lobule. Since there was no intervention in the waitlist group for CID patients, the elevated GBC values observed in this group may reflect the natural progression of CID or a nonspecific effect. Notably, correlation analysis showed a negative correlation between the acupuncture and waitlist groups in the spatial pattern of changes in GBC values (r = − 0.43, p_spin_ = 0.0034). The elevated GBC values in the waitlist group contrasted with the decrease in GBC values induced by acupuncture treatment, implying that there were different patterns of GBC changes between the two groups. The above findings suggest that acupuncture therapy may affect the activity of specific brain areas by regulating biological processes associated with inflammation, thereby improving the clinical symptoms of CID.

Additionally, we recognize that excluding a sham arm limits our ability to disentangle specific needling effects from contextual/placebo contributions. Convergent evidence suggests these comparators are not interchangeable: a coordinate-based fMRI meta-analysis [6] in primary insomnia reported robust modulation of default-mode/fronto-cortical regions with true acupuncture, while indicating that non-acupoint (sham) stimulation can still yield measurable frontal effects—patterns distinct from no-treatment comparators. Likewise, a clinical study [30] showed clinical improvements for true acupuncture that exceed sham and waitlist, but also demonstrates that sham can produce non-trivial benefits relative to waiting, underscoring a physiological placebo component. On the inflammatory side, a systematic review [61] indicated that auricular stimulation can reduce peripheral cytokines (e.g., IL-6, TNF-α), whereas the magnitude and consistency of such changes under sham conditions remain smaller or less reliable than under active treatment—again implying differential biological engagement beyond expectancy alone. Considering this literature, we acknowledge that our waitlist-controlled design cannot fully separate acupuncture-specific effects from contextual influences, and we plan future sham-controlled trials with cytokine sampling and neuroimaging across all arms to address this limitation.

### Transcriptomic signatures of GBC changes after acupuncture treatment

In this study, we not only observed changes in functional connectivity of key brain regions induced by acupuncture treatment but also revealed the microscopic mechanism underlying these macroscopic functional connectivity changes at the molecular and cellular levels. By utilizing the PLS-R algorithm, we established associations between macroscopic imaging phenotypes and microscopic gene expression. We found that GBC showed a tendency to decrease after acupuncture treatment, especially in regions where genes with negative weights in the PLS1-gene set were highly expressed. These genes, such as TNAK, can influence several immune and inflammation-related signal transduction pathways [46], including the NF-κB and interferon signaling pathways, which are closely related to inflammation.

Further cell type enrichment analysis showed that the reduction in GBC values after acupuncture treatment was associated with high astrocyte expression. Astrocytes, one of the most abundant cell types in the central nervous system, play important roles in neuroinflammation, neuroprotection, and maintaining brain microenvironment stability [62]. During neuroinflammatory states, astrocytes become “activated” and express a variety of inflammatory factors such as IL-1β, TNF-α, and others [63]. These factors and substances are key components of inflammatory signaling pathways, such as the NF-κB signaling pathway, which acupuncture treatment has the potential to modulate. For example, acupuncture has been found to inhibit the activation of the NF-κB signaling pathway, potentially reducing the production of inflammatory factors [64]. Additionally, acupuncture promotes the release of neuroprotective factors, such as brain-derived neurotrophic factor [65], which may help to inhibit the overactivation of astrocytes, thereby mitigating neuroinflammation. We also found significant enrichment of several GO terms broadly associated with neuroinflammation. Such as the activation of the innate immune response, mast cell secretion of histamine, and mature B cell differentiation. These findings suggest that the decrease in GBC induced by acupuncture therapy is closely related to its effects on modulating neuroinflammatory processes.

### Neuroinflammation-informed GBC-transcriptomic signatures associated with reduction in peripheral pro-inflammatory factors after acupuncture treatment

Our study identified that brain regions with reduced GBC, such as the pallidum and prefrontal cortex, are commonly associated with neuroinflammatory processes in their brain region functions [55]. This may be due to the reduction in pro-inflammatory factors after acupuncture treatment, leading to decreased transmission of inflammatory signals between these local brain regions and other parts of the brain. Guided by the selective GBC decreases in neuroinflammation-susceptible regions (pallidum, frontal pole, orbitofrontal cortex) and the positive coupling between our GBC-transcriptomic signature and reductions in IL-6, we hypothesize that acupuncture may down-modulate neuroinflammation—particularly astrocyte-enriched processes—which subsequently may normalize large-scale functional connectivity and alleviates insomnia. Nonetheless, we acknowledge that the reverse pathway, in which changes in brain connectivity drive alterations in peripheral inflammation, remains plausible. To disentangle this bidirectional relationship, future animal studies could selectively block neuroinflammation (e.g., inhibiting astrocyte or microglial NF-κB signaling or neutralizing cytokines) during acupuncture to test whether GBC and behavioral improvements are abolished, or induce inflammation (e.g., via IL-6 infusion) to determine if abnormal connectivity re-emerges despite acupuncture [63]. Furthermore, chemogenetic or optogenetic manipulation of astrocytes or microglia combined with fMRI or fiber photometry, along with dense time-course measurements of brain connectivity and CSF/serum cytokines, could clarify the temporal and causal sequence linking neuroimmune modulation to network changes [66].

Several limitations need to be considered. First, our sample size was moderate, which may limit our ability to detect small effects after performing multiple comparisons across 83 brain regions. A larger cohort study is needed to confirm our findings. Second, although the use of a waitlist control enabled observation of acupuncture effects under real-world conditions and accounted for the natural course of insomnia, it limited our ability to fully distinguish specific effects of acupuncture from placebo-related factors. Future studies should consider incorporating sham controls to further validate our findings. Third, our study relied on self-reported measures such as the PSQI and ISI scores to assess clinical outcomes. Incorporating objective sleep measurements, such as polysomnography, in future studies would provide more robust evidence on the effects of acupuncture on sleep quality. Fourth, peripheral cytokines were collected only in the acupuncture arm to minimize invasive procedures and participant burden in participants not receiving active treatment. Future sham-controlled trials with cytokine sampling across all study arms are warranted to allow definitive between-group comparisons of inflammatory trajectories. Finally, although imaging transcriptomics enhances our understanding of macroscopic neuroimaging phenotypes, the associations presented are potential interpretations rather than direct molecular evidence. Future research should consider in-depth animal studies to further validate the microbiological targets identified in this study.

## Conclusion

In summary, we found that acupuncture alleviated insomnia symptoms, reduced peripheral inflammatory markers such as IL-6, and decreased GBC in key regions implicated in neuroinflammation. Notably, reductions in GBC following acupuncture treatment were associated with astrocyte-related neuroinflammatory processes. These neuroinflammation-informed GBC-transcriptomic signatures were further supported by their significant correlation with decreases in IL-6 levels after treatment. Together, these findings provide evidence for the cross-scale mechanisms underlying the effects of acupuncture in patients with chronic insomnia.

## Supplementary Information

Supplementary file 1.

## Data Availability

The datasets generated and analyzed during the current study are not publicly available due to institutional ethics committee restrictions and participant privacy considerations. However, de-identified data supporting the findings are available from the corresponding authors upon reasonable request.

## Abbreviations

CID: Chronic insomnia disorder
rs-fMRI: Resting-state functional magnetic resonance imaging
GBC: Global brain connectivity
IL-6: Interleukin-6
mPFC: Medial prefrontal cortex
AHBA: Allen human brain atlas
PLS-R: Partial least squares regression
PLS1: The first component in PLS-R
DK: Desikan-Killiany
