# Supplementary material for: Neuroinflammation-informed neuroimaging-transcriptomic signatures explaining acupuncture’s therapeutic effects in chronic insomnia
Source: Chin Med. 2025 Nov 28;20:207. doi: 10.1186/s13020-025-01236-5 (PMC12661874; doi:10.1186/s13020-025-01236-5)
Supplement: Supplementary file 1 [file 13020_2025_1236_MOESM1_ESM.docx]

**Supplementary materials**

**Decoding GBC changes from a molecular perspective**

**Estimation of microarray gene expression maps**

Gene expression data were extracted from the AHBA ^1^. The AHBA gene expression data were processed utilizing the abagen toolbox ^2^ (version 0.1.3; https://github.com/rmarkello/abagen), which included steps such as filtering of microarray probes based on intensity, selecting a single probe per gene, matching samples to brain regions as defined by the Desikan-Killiany 83 atlas ^3^, normalization, and data aggregation both within and across parcellations. The final gene expression data were represented by an 83 × 15,633 matrix for each donor, correlating brain regions to the retained genes. Genes with low similarity across donors (r < 0.2) were excluded, resulting in a final analysis of 12,506 genes. Due to the availability of right hemisphere data for only two donors, the transcriptomic-imaging association analysis was limited to the left hemisphere, encompassing 34 cortical and 7 subcortical regions, and was represented by a 41-region × 12,506-gene matrix.

**PLS regression relating gene expression and GBC alterations**

Partial least squares regression (PLS-R) was utilized to explore the relationship between brain gene expression and changes in GBC induced by acupuncture ^4^. This method ranks genes based on the multivariate spatial correlation between their expression profiles and GBC alterations. The primary component of PLS, known as PLS1, represents the linear combination of gene expressions, each weighted to maximize alignment with the GBC change pattern. PLS1 aims to maximize the covariance between GBC alterations and gene expression profiles, but it does not necessarily explain the highest variance in GBC changes. With an increase in the number of components analyzed (from 1 to 15) ^5^, a diminishing variance explanation for GBC changes was observed in our study. Therefore, we focused on PLS1, which accounted for the most variance, in subsequent analyses. Bootstrapping (resampling with replacement of the 41 brain regions 10,000 times) was used to estimate the variability of each gene’s PLS1 weight. The ratio of the PLS1 weight of each gene to its bootstrap standard error was used to calculate the Z scores and, hence, rank the genes according to their contribution to PLS1 ^6^.

**Gene enrichment analysis**

To explore the pivotal biological processes and cell types associated with acupuncture-induced changes in GBC, we applied a gene category enrichment analysis tool (available at <https://github.com/benfulcher/GeneCategoryEnrichmentAnalysis>) ^7^. Biological process categories were associated with specific subsets of genes as annotated in the Gene Ontology (geneontology.org). For each category, a score was calculated as the mean gene loading within the category, with the gene loading defined as the Z score obtained from the bootstrapping procedure. A null-spatial model was created by permuting the response variables 10,000 times while maintaining spatial autocorrelation. For each permutation, PLS analysis was re-executed with the original gene expression data and the permuted response variables, enabling the recalculation of null gene loadings and GO category scores. The significance p-value for the null-spatial model (p_spin_) for each GO category was calculated based on the proportion of permuted scores that are more extreme than the observed category score.

Cell-type enrichment analysis was conducted using cell-specific aggregate gene sets ^8^. By implementing hierarchical clustering of regional topographies from all cell types specific to the studies in the AHBA, we delineated seven principal canonical cortical cell classes, specifically astrocytes, endothelial cells, microglia, excitatory neurons, inhibitory neurons, oligodendrocytes, and oligodendrocyte precursors ^8^. For each cell type, the proportion of its genes in the GBC change-related gene set was initially calculated. An equal number of genes were randomly selected from the AHBA’s 12,506 genes 10,000 times to construct a null model, facilitating subsequent comparisons with the observed proportions for each cell type. For each cell type, both under-representation and over-representation conditions are considered. Since multiple cell types are being tested (n = 7), FDR correction was used to correct multiple comparisons.

**Spatial Null Models**

To validate the statistical significance of the gene-neuroimaging correlations revealed by the PLS-R models, we compared the real-data-derived PSL1 against null distributions. These distributions were generated from 10,000 spatial permutations (the "spin test"). This permutation involved randomly rotating the neuroimaging vector rows based on a spherical projection of the cortical surface ^9^. The spatial permutation technique is available at <https://github.com/frantisekvasa/rotate_parcellation>. This method ensures the preservation of the inherent correlational structure of the cortical surface data, offering stringent control against false positives in contrast to conventional random permutation tests ^9^. This model was used to facilitate the examination of the null PLS1 variance against the actual PLS1 variance derived from the original data. A PLS1 explained variance exceeding the 95th percentile in the distribution of the explained variance from the spatially rotated (p_spin_) null models was considered statistically significant. Since subcortical regions lack a spherical structure, we just randomly rotated subcortical values 10,000 times to generate a null model. Additionally, for the correlation analysis between two cortical maps, we also conducted spatial-null models to reduce spatial autocorrelations. This method compares the empirical correlation between two spatial maps to a set of null correlations. Each correlation between two cortical maps is reported with a p-value from spherical permutation (p_spin_), derived from comparing the actual Spearman Rho against a null distribution of 10,000 correlations, using one real map and rotated projections of the other.

## Table S1. Demographics of six adult donors in the AHBA dataset.

| Donor | Number of cohorts | Age | Sex | Ethnicity | Post-mortem  interval **a** |
| --- | --- | --- | --- | --- | --- |
| H0351.2001**b** | 946 | 24 | Male | African  American | 23h |
| H0351.2002**b** | 893 | 39 | Male | African  American | 10h |
| H0351.1009 | 363 | 57 | Male | Caucasian | 25.5h |
| H0351.1012 | 529 | 31 | Male | Caucasian | 17.5h |
| H0351.1015 | 470 | 49 | Female | Hispanic | 30h |
| H0351.1016 | 501 | 55 | male | Caucasian | 18h |

Notes: **^a^** Post-mortem interval is defined as the time period from the time of death to the time the tissue is frozen. **^b^** These donors have tissue cohorts collected across the left and right hemispheres, while the other donors have tissue cohorts only in the left hemisphere.

**Table S2.** Two groups on regional global brain connectivity (GBC)

| grouping | Region | MNI coordinates (x, y, z) | | | t-statistic | *p*-value |
| --- | --- | --- | --- | --- | --- | --- |
| acupuncture | L_ fusiform gyrus | -7.66 | -23.54 | -92.31 | -2.14 | 0.044 |
|  | L_ Inferior temporal gyrus | -41.78 | -19.01 | -83.63 | -2.23 | 0.036 |
|  | L_ isthmus cingulate cortex | 85.56 | -34.29 | -29.18 | -2.36 | 0.028 |
|  | L_ rostral middle frontal gyrus | 8.89 | 88.97 | 33.13 | -2.27 | 0.033 |
|  | L_ frontal pole | 40.80 | 90.84 | -6.46 | -3.18 | 0.004 |
|  | R_ lateral orbitofrontal cortex | 0.99 | 88.91 | -38.68 | -2.17 | 0.041 |
|  | R_ pars opercularis (of the inferior frontal gyrus) | 71.43 | 63.44 | 18.56 | 2.14 | 0.044 |
|  | R_ supramarginal gyrus | 85.68 | -33.31 | 16.99 | 3.16 | 0.005 |
|  | R_ insula | 63.09 | 56.75 | -42.30 | 2.51 | 0.020 |
|  | L_ globus pallidus | / | / | / | -3.52 | 0.002 |
| Waitlist | L_ paracentral lobule | 70.55 | -26.79 | 62.10 | 2.11 | 0.048 |
|  | R_ isthmus cingulate cortex | -94.45 | -14.26 | -11.65 | 2.28 | 0.034 |
|  | R_ parahippocampal Gyrus | -49.27 | 10.23 | -84.17 | 2.19 | 0.041 |

Note: The table provides detailed information for three statistically significant regions, including anatomical labels, coordinates (in fsaverage, MNI305 space), paired t-test t-statistics, and p-values. All p-values are <0.05, no region survived after FDR correction. L, Left; R–Right.

**Table S3**. Brain cell-type ensemble enrichment – transcriptomic vulnerability to connectivity changes after acupuncture treatment

| Brain cell-type | ER | p_FDR_ |
| --- | --- | --- |
| Astrocytes | 0.065 | 0.033 |
| Microglia | 0.061 | 0.244 |
| OPC | 0.033 | 0.722 |
| Neuro_Exc | 0.015 | < 0.001 |
| Neuro_Inb | 0.026 | 0.118 |
| Oligodendrocytes | 0.011 | 0.328 |
| Endothelial | 0.059 | 0.092 |

Note: Negative enrichment rations indicate enrichment for a certain brain cell-type among genes with negative weights; the reverse applies to positive enrichment ratios. Abbreviations: ER, enrichment ratio; Exc, excitatory; In, inhibitory; OPC, oligodendrocyte precursor cells.

**Table S4**. Gene ontology (biological pathways) ensemble enrichment – transcriptomic vulnerability to connectivity changes after acupuncture treatment

| Description | Category Scores | p_FDR_ |
| --- | --- | --- |
| activation of innate immune response | -4.144 | <0.001 |
| histamine secretion by mast cell | -4.077 | <0.001 |
| inner cell mass cell fate commitment | -3.806 | <0.001 |
| mature B cell differentiation | -3.621 | <0.001 |
| natural killer cell proliferation | -3.569 | <0.001 |
| histone monoubiquitination | -3.525 | <0.001 |
| negative regulation of blood vessel remodeling | -3.502 | <0.001 |
| exocytosis | -3.460 | <0.001 |
| positive regulation of neurotransmitter secretion | -3.394 | <0.001 |
| mast cell chemotaxis | -3.248 | <0.001 |
| response to molecule of bacterial origin | -3.192 | <0.001 |
| regulation of asymmetric cell division | -3.133 | <0.001 |
| peripheral nervous system development | -3.059 | <0.001 |
| regulation of cell cycle | -3.020 | <0.001 |

Note: Negative enrichment rations indicate enrichment for a certain category among genes with negative weights. The category score was calculated as the mean gene loading (with loading for a gene being a Z score in the bootstrapping procedure) within the category.

**References**

1. Hawrylycz MJ, Lein ES, Guillozet-Bongaarts AL, et al. An anatomically comprehensive atlas of the adult human brain transcriptome. *Nature*. 2012;489(7416):391-399. doi:10.1038/nature11405

2. Markello RD, Arnatkeviciute A, Poline JB, Fulcher BD, Fornito A, Misic B. Standardizing workflows in imaging transcriptomics with the abagen toolbox. *eLife*. 2021;10:e72129. doi:10.7554/eLife.72129

3. Desikan RS, Ségonne F, Fischl B, et al. An automated labeling system for subdividing the human cerebral cortex on MRI scans into gyral based regions of interest. *NeuroImage*. 2006;31(3):968-980. doi:10.1016/j.neuroimage.2006.01.021

4. Morgan SE, Seidlitz J, Whitaker KJ, et al. Cortical patterning of abnormal morphometric similarity in psychosis is associated with brain expression of schizophrenia-related genes. *Proc Natl Acad Sci*. 2019;116(19):9604-9609. doi:10.1073/pnas.1820754116

5. Martins D, Dipasquale O, Veronese M, et al. Transcriptional and cellular signatures of cortical morphometric remodelling in chronic pain. *Pain*. 2022;163(6):e759-e773. doi:10.1097/j.pain.0000000000002480

6. Whitaker KJ, Vértes PE, Romero-Garcia R, et al. Adolescence is associated with genomically patterned consolidation of the hubs of the human brain connectome. *Proc Natl Acad Sci*. 2016;113(32):9105-9110. doi:10.1073/pnas.1601745113

7. Fulcher BD, Arnatkeviciute A, Fornito A. Overcoming false-positive gene-category enrichment in the analysis of spatially resolved transcriptomic brain atlas data. *Nat Commun*. 2021;12(1):2669. doi:10.1038/s41467-021-22862-1

8. Seidlitz J, Nadig A, Liu S, et al. Transcriptomic and cellular decoding of regional brain vulnerability to neurogenetic disorders. *Nat Commun*. 2020;11(1):3358. doi:10.1038/s41467-020-17051-5

9. Váša F, Seidlitz J, Romero-Garcia R, et al. Adolescent Tuning of Association Cortex in Human Structural Brain Networks. *Cereb Cortex*. 2018;28(1):281-294. doi:10.1093/cercor/bhx249
